# Cross-cultural validation of the IRB Researcher Assessment Tool: Chinese Version

**DOI:** 10.1186/s12910-021-00699-z

**Published:** 2021-09-28

**Authors:** Xing Liu, Ying Wu, Min Yang, Yang Li, Jessica Hahne, Kaveh Khoshnood, Linda Coleman, Xiaomin Wang

**Affiliations:** 1 Center for Clinical Pharmacology, The Third Xiangya Hospital, Central South University, Changsha, 410013 Hunan People’s Republic of China; 2 Medical Ethics Committee, Xiangya Hospital of Central South University, 87 Xiangya Road, Changsha, 410008 Hunan People’s Republic of China; 3 School of Public Administration, Central South University, Changsha, 410075 Hunan China; 4 Xiangya School of Nursing, Central South University, Changsha, 410013 Hunan China; 5 Yale School of Public Health, Yale University, 60 College Street, New Haven, CT 06510 USA; 6 Human Research Protection Program, Yale University, 150 Munson Street, New Haven, CT 06511 USA

**Keywords:** China, Institutional Review Board, Cross-cultural validation

## Abstract

**Background:**

Using an effective method for evaluating Institutional Review Board (IRB) performance is essential for ensuring an IRB’s effectiveness, efficiency, and compliance with applicable human research standards and organizational policies. Currently, no empirical research has yet been published in China evaluating IRB performance measures by the use of a standardized tool. This study was therefore conducted to develop a Chinese version of the IRB Researcher Assessment Tool (IRB-RAT), assess the psychometric properties of the Chinese version (IRB-RAT-CV), and validate the tool for use in China.

**Methods:**

In this cultural adaptation, cross-sectional validation study, the IRB-RAT-CV was developed through a back-translation process and then distributed to 587 IRB staff members and researchers in medical institutions and schools in Hunan Province that review biomedical and social-behavioral research. Data from the 470 valid questionnaires collected from participants was used to evaluate the reliability, content validity, and construct validity of the IRB-RAT-CV.

**Results:**

Participants’ ratings of their ideal and actual IRB as measured by the IRB-RAT-CV achieved Cronbach's alpha 0.989 and 0.992, Spearman-Brown coefficient 0.964 and 0.968, and item-total correlation values ranging from 0.631 to 0.886 and 0.743 to 0.910, respectively.

**Conclusion:**

The IRB-RAT-CV is a linguistically and culturally applicable tool for assessing the quality of IRBs in China.

## Background

Institutional Review Boards (IRBs), also known as Research Ethics Committees (RECs), are formally designated to review, approve, and monitor research [1]. IRBs have the responsibility to ensure ethical protection of human research participants, compliance with applicable regulatory requirements, and adherence to other human research standards [2]. To avoid unnecessary delays in the research process, an IRB should perform its review efficiently and in a timely manner. IRBs should develop and evaluate mechanisms to ensure that their operations are transparent, accountable, consistent, and of high quality [3]. Strengthening ethical review is critical to maintaining the public’s trust in research. Review procedures should be standardized, to decrease the time needed for review and enable research to occur without unnecessary delays [4].The quality of biomedical research also depends on high-quality review by IRBs. IRBs function as gatekeepers, promoting ethical research by allowing only ethically approved studies to move forward [5].

Concerns have been expressed regarding the adequacy of ethics review systems in developing countries [6–8]. IRBs in developing countries are hindered by a number of factors including inadequate training of members, lack of member diversity, and scarcity of resources [9–12]. Evaluating the effectiveness of an IRB continues to be a difficult task [13]. Therefore, institutions in developing countries that are engaged in human subjects research urgently need an effective and reliable tool to evaluate the quality of IRBs. This is especially true in China, where the role and importance of IRBs is rapidly expanding, but where recent research indicates IRBs routinely face problems related to basic performance, including absence of supervision, vague review criteria, limitations of ethics committee competence, inadequate knowledge of ethics, and poor tracking of reviews [14]. Since 2016, the National Health Commission (NHC) and National Medical Product Administration (NMPA)—formerly the China Food and Drug Administration (CFDA)—have stipulated that medical and health institutions in China should regularly evaluate the quality and other performance measures of their IRBs [15]. After joining the International Council for Harmonization of Technical Requirements for Pharmaceuticals for Human Use (ICH) in 2017, the NMPA established certain rules and issued a series of documents focused on the promotion and development of clinical research and IRBs. For example, on October 8, 2017, it proposed several changes to improve both the operating mechanism and the efficiency of IRBs. This included implementing new IRB models in the form of regional IRBs and authorized external IRBs to conduct ethical review of clinical trials. In addition, if a multi-center clinical trial is carried out within China and is approved by the IRB at a lead institution, other participating institutions are now permitted to recognize the approval of the lead institution and are not required to duplicate IRB review of the study at the respective local institutions [16].

In recent years, the scope and role of IRBs in China has continued to expand. As of 2018, the drug regulatory department under the State Council proposed the implementation of implied licensing for drug clinical trials. The drug regulatory department must decide whether or not to approve a clinical trial sponsor within 60 working days from the date of accepting the clinical trial application. If the notification of approval is not received within the time limit, approval is granted by default. IRBs are thus given flexibility to conduct an ethical review within 60 days of the application for clinical trial consent or after 60 days [17]. Building on this change, on August 26, 2019, a mechanism was established that enabled IRBs at drug clinical trial institutions to register on an NMPA platform granting them authority to review drug clinical trials [18, 19]. And IRBs have taken on greater responsibilities as stipulated by law in China: the Law of the People's Republic of China on Basic Medical and Health Care and Health Promotion in 2019 [20] and The Civil Code in 2020 established at the legal level for the first time that all research must be reviewed and approved by IRBs [21]. As the role of IRBs grows in China, there is an urgent need for the development of IRB evaluation tools that can be used to identify shortcomings and improve IRB performance.

Previous studies evaluating IRB performance have been conducted in several countries and regions, including the United States [13], Egypt [9], the Middle East [22], Africa [23], India [24], Thailand [25], Myanmar [26], and Pakistan [27]. Although some of this research involved evaluation using validated tools (US [28, 29], India [30], Peru [31], and Singapore [32]), most research of this type has involved use of self-developed survey instruments within individual institutions. To our knowledge, only one evaluation study of an IRB in China has formerly been published—a self-developed evaluation conducted by members of this research team [33].

One of the more commonly used validated tools for IRB evaluation is the IRB Researcher Assessment Tool (IRB-RAT), developed by Keith-Spiegel and Koocher in 2005. The purpose of the tool is to evaluate an IRB’s function and activities [34]. The IRB-RAT can be used to assess the perceptions of investigators and IRB members regarding the relative importance of different aspects of IRB performance, as well as the degree to which those different aspects are in need of improvement [34]. Reeser et al. utilized the IRB-RAT to gain insight into the ways in which Marshfield Clinic Research Foundation’s IRB was perceived by those who routinely interacted with the Office of Research Integrity and Protections [28]. Afterwards, Tiffany et al. used the IRB-RAT to assess how investigators and IRB members at a hospital in India viewed various aspects of their IRB [30]. Daniel et al. used the IRB-RAT to guide quality improvement of IRBs [29]. Roque-Henriquez et al. translated the tool into Spanish and demonstrated that their version of the IRB-RAT has sufficient reliability and validity in Peru [31]. Labude et al. used the IRB-RAT to ascertain general views regarding the function and characteristics of a biomedical research IRB in Singapore [32].

The cultural environment, local context, and ethical norms within each country in which an IRB exists may affect how the IRB is assessed [30]. Due to the growing scope and role of IRBs in China, there is a need for further IRB evaluation studies using culturally appropriate, validated tools. Although the IRB-RAT is a tool to measure IRB performance, it was developed within the Western cultural context, and it is not known to what degree it is suitable for IRB evaluation in China. Currently, there is no validated Chinese translation of the IRB-RAT available and no published use of the IRB-RAT in China. The aim of this study is therefore to create an adapted version of the IRB-RAT tool for use in China (IRB-RAT-CV) and to evaluate its reliability and validity.

## Methods

### Study design

This cultural adaptation and cross-sectional validation study was conducted from June 2020 to June 2021 in Hunan Province, Central Southern China. We translated and adapted the IRB-RAT to develop the IRB-RAT-CV, then distributed the IRB-RAT-CV and a demographic questionnaire, through convenience sampling, to 587 IRB employees and researchers in medical institutions and schools in Hunan Province.

### The IRB-RAT instrument

The IRB-RAT is a self-report measure of IRB performance [35]. It lists 45 items describing IRB functions and activities. The questionnaire was designed to assess the relative importance to the respondents of the items, clustered into eight themes. Themes include: procedural justice (how the decision-making process is carried out); absence of bias (a feature of procedural justice); pro-science sensitivity and commitment; interactional justice (interpersonal sensitivity and justification); formalities (an IRB’s formal functioning, structure, and composition); upholding of rights of human research participants; IRB outreach (offering services beyond those mandated); and competence (how competently the IRB performs its functions). Respondents are asked to give two Likert Scale ratings on each item, to indicate both the importance of that item within their conception of an ideal IRB, and how closely the item describes their actual IRB. Specifically, the survey asks, “As an investigator, how important is each one (item) to you in your work? First rate how important each item would be to you to do your best work along a 7-point continuum with 7 = ‘Absolutely essential’ to 1 = ‘Not important.’ Then, rate how well that item describes your actual IRB on the same item, with 7 = ‘Highly descriptive’ to 1 = ‘Not at all descriptive.’”.

By comparing respondents’ ideal and actual IRB ratings, the IRB-RAT creates a performance standard for the evaluation of each IRB activity or function [29]. The sums of all respondents’ ideal and actual IRB ratings are each calculated to determine a total ideal IRB score and a total actual IRB score. A higher total actual IRB score is considered to indicate higher quality of performance of the IRB being evaluated. The total actual IRB score is also compared to the total ideal IRB score as a measure of IRB performance.

### Translation of the IRB-RAT

The IRB-Researcher Assessment Tool (IRB-RAT) developed by Keith-Spiegel and Koocher in 2005 includes two versions (Version A: double pass, Version B: single pass). We chose to translate and adapt Version B for the purpose of our study, for the following reasons: Version B is shorter and thus more feasible for participants to complete; the process for discerning and calculating difference scores and identifying trends in themes is simpler for Version B [34]; and Version B can simultaneously measure participants’ perceptions of an ideal IRB and their actual IRB. Permission to translate the IRB-RAT was obtained from Keith-Spiegel and Koocher, the original designers. We adopted Brislin’s translation model for cross-cultural translation, which uses back-translation [36]. First, two researchers (Y.W. and X.M.W.) drafted the IRB-RAT-CV. Both researchers are bilingual translators, with records of passing scores on the highest-tier college-level English test in China (CET-6). After that, an ethics expert in China proofread the draft. Next, two university English Department faculty members from the same institution who were previously unfamiliar with the IRB-RAT-CV translated it back into English. We sent the back-translated-English version to Koocher and integrated their feedback. Finally, the two translators (Y.W. and X.M.W.) compared the Chinese version with the original English version to identify any linguistic inaccuracies.

In addition, the IRB-RAT-CV developed in our study underwent three rounds of collaborative review by eight experts with diverse roles (two IRB members, two IRB staff members, two IRB managers, and two investigators) to check whether the items in the questionnaire were related to IRB review in China, and whether the content of the questionnaire was consistent with a Chinese cultural viewpoint. No major issues concerning cultural adaptation were reported by the Chinese experts. The questionnaire was also evaluated and revised for fluency, readability, and comprehension by a panel of IRB office staff and students with IRB-related experience. The final IRB-RAT-CV was then distributed to the study participants along with an anonymous demographic questionnaire.

### Participants and sampling method

We used convenience sampling to distribute electronic questionnaires to 587 IRB employees and researchers in medical institutions and schools in Hunan Province that review biomedical research and social-behavior research. The sample size for this study was calculated according to internationally accepted principles whereby sample size required for scale development and validation is usually 5–10 times the total number of items [37]. Because there are 45 items in the IRB-RAT, the number of participants required was between 225 and 450. After factoring in the number of invalid questionnaires received (see “Results” section), the final required sample size was estimated to be 270–540 participants.

Participants were recruited using WeChat, a popular messaging app in China. Our research team initiated a chat group on WeChat, inviting IRB members, staff, and researchers from 51 different institutions in Hunan Province. The online questionnaire for our study, accessible through a web link, was distributed by sending the link to this group through WeChat and asking group members to forward the link to additional colleagues in their work departments.

The first part of the online questionnaire consisted of an informed consent form that included the purpose, risks, and benefits of the study. Participants who consented on the first page were then presented with a demographic questionnaire and the IRB-RAT-CV survey. Participation was voluntary and anonymous. No personally identifiable information was collected. IRB employees including IRB chairs, vice chairs, members, and staff were surveyed. We also included researchers who had undertaken at least one or more biomedical research projects involving human participants.

### Data analysis

Using SPSS 26.0 (IBM, Chicago, IL, USA) with AMOS 24.0, data were analyzed to assess the reliability and validity of the IRB-RAT-CV. Details of the researchers’ and IRB staff members’ responses to the IRB-RAT-CV will be presented elsewhere.

Before performing data analysis, the data were checked for missing values and the missing values were replaced using multiple imputation. The reliability of the IRB-RAT-CV was assessed using Cronbach’s alpha, split-half reliability (Spearman–Brown coefficient), and item-to-total correlation coefficients. A Cronbach’s alpha coefficient greater than 0.7 indicates that the internal consistency of the instrument is very good [38]. Content validity was measured using Kendall's W coefficient of concordance, where Kendall's W = 0.8–1.0 would be considered excellent, Kendall's W = 0.6–0.8, 0.4–0.6 would be moderate, Kendall's W = 0.2–0.4 would be fair, and Kendall’s W = 0.0–0.2 would be poor [39]. We checked the fit between the factor structure and the data using confirmatory factor analysis (CFA). We chose the method of maximum likelihood (ML) for the CFA, and items with factor loading above 0.40 were used for factor interpretation [40]. In this study, RMSEA, CFI, TLI, and NFI were used as fitting indices. By these metrics, a good fit is indicated by a normed chi-square (χ^2^/df) value of 0.0–3.0; root mean square error of approximation (RMSEA) value of 0–0.05 (good fit) or 0.05–0.1 (satisfactory fit); and by comparative fit index (CFI), Tucker Lewis index (TLI), and normed fit index (NFI) values greater than 0.90.

## Results

### Demographic characteristics

Of the 587 questionnaires returned, 117 were determined to be invalid because they were completed by professionals not working directly within the IRBs or not doing research directly evaluated by the IRBs at the institutions included in our study. Of the remaining 470 valid questionnaires, 176 were completed by IRB staff, 277 by researchers, and 17 by respondents who were both IRB-related staff and researchers. As shown in Table 1, the mean age of the respondents was 39.86 ± 9.22 years, and the majority (60.4%) were female. The education levels of participants were as follows: bachelor's degree and below (22.3%), master's degree (44.5%), and PhD (33.2%). A small number of participants (22.8%) reported having studied abroad for more than three months. The average time respondents had been in the workforce was 8.41 ± 6.94 years. Most participants (79.4%) reported that the IRB they worked for had an independent ethics committee office to coordinate administrative tasks, and most participants (83.2%) reported having received ethics training in the previous three years.

**Table 1:** Characteristics of participants

Characteristics		Total (N = 470)
Age (mean ± SD, years)		39.86 ± 9.22
Participants (%)	IRB staff	176 (37.5%)
	Researcher	277 (58.9%)
	Both IRB-related staff and researcher	17 (3.6%)
Sex (%)	Male	186 (39.6%)
	Female	284 (60.4%)
Average time in the workforce (mean ± SD, years)		8.41 ± 6.94
Education level (%)	Bachelor's degree or below	105 (22.3%)
	Master's degree	209 (44.5%)
	Ph.D	156 (33.2%)
Studied abroad for more than 3 months (%)	Yes	107 (22.8%)
	No	363 (77.2%)
Received ethics training in the last 3 years (%)	Yes	391 (83.2%)
	No	79 (16.8%)
Works at an IRB that has an independent ethics committee office (%)	Yes	373 (79.4%)
	No	59 (12.5%)
	Do not know	38 (8.1%)
Professional title	Primary level professional title	56 (11.9%)
	Intermediate level professional title	155 (33%)
	Associate Professor	119 (25.3%)
	Professor	96 (20.4%)
	None	44 (9.4%)
Institution	Tertiary (level 3) hospital	392 (83.4%)
	Secondary (level 2) hospital	13 (2.8%)
	Medical school	46 (9.8%)
	Life Medicine Research Institute	5 (1%)
	Other (law office, colleges, community, centers for disease control and prevention)	14 (3%)

SD = standard deviation; IRB = institutional review board

### Consistency and reliability analysis

The Cronbach's alpha coefficients for the ideal IRB and actual IRB ratings of participants in the IRB-RAT-CV were 0.989 and 0.992, and the split-half reliability (Spearman-Brown coefficient) were 0.964 and 0.968, respectively. The Cronbach's alpha coefficients for each factor ranged from 0.894 to 0.957 and 0.927 to 0.970, and the split-half reliability for each factor ranged from 0.890 to 0.950 and 0.933 to 0.957, respectively. These findings indicate good internal consistency and reliability. The item-total correlation ranged from 0.631 to 0.886, and 0.743 to 0.910, which also indicated good correlation between each item and the overall IRB-RAT-CV.

### Validity

#### Content validity

The degree of coordination of experts was measured by Kendall's W, which has a value between 0.0 and 1.0. The closer the value is to 1.0, the better the coordination of all experts on the ratings of all entries, and vice versa. Lower scores suggest greater inconsistency among experts in their perceptions of the relative importance of each entry. The results of our analysis showed that Kendall’s W was 0.410 (*p* < 0.001), indicating that experts' opinions converged to a moderate degree.

#### Construct validity

CFA was used to assess the construct validity of the IRB-RAT-CV. All 470 valid questionnaires were selected for the CFA of ML. As shown in Tables 2 and 3, the factor loadings of the ideal IRB and actual IRB ratings were significant and greater than 0.6, and the factor structure fit the data well. We modified the models twice and achieved better indicators. As shown in Table 4, results were as follows for the ideal IRB: χ^2^/df = 2.811, RMSEA = 0.062, NFI = 0.904, TLI = 0.931, CFI = 0.936; and for the actual IRB: χ^2^/df = 2.967, RMSEA = 0.065, NFI = 0.914, TLI = 0.936, CFI = 0.941; indicating a good model fit.

**Table 2:** Standardized Factor Loadings and error variances of 45-Item IRB-RAT-CV for Ideal IRB

Ideal IRB			
Domain	Items	Factor-item loading	Error variances
Procedural justice	Item 1*	0.69	0.34
	Item 2*	0.85	0.15
	Item 3	0.83	0.16
	Item 4	0.88	0.12
	Item 5	0.87	0.13
	Item 6	0.84	0.16
	Item 7	0.87	0.12
Interactional justice	Item 8	0.88	0.13
	Item 9	0.84	0.19
	Item 10	0.89	0.12
	Item 11	0.87	0.15
	Item 12	0.87	0.15
Absence of bias	Item 13	0.88	0.13
	Item 14	0.92	0.08
	Item 15	0.87	0.13
	Item 16	0.92	0.08
	Item 17	0.84	0.16
Pro-science sensitivity	Item 18	0.88	0.12
	Item 19	0.84	0.17
	Item 20	0.81	0.23
	Item 21	0.86	0.15
	Item 22	0.85	0.15
IRB competence	Item 23	0.85	0.14
	Item 24	0.90	0.09
	Item 25	0.90	0.10
	Item 26	0.89	0.11
	Item 27	0.91	0.10
	Item 28	0.81	0.22
	Item 29	0.75	0.35
	Item 30	0.88	0.12
IRB outreach	Item 31	0.88	0.13
	Item 32	0.90	0.13
	Item 33	0.91	0.12
	Item 34	0.83	0.24
IRB formal functioning, structure, and composition	Item 35	0.91	0.10
	Item 36	0.91	0.10
	Item 37	0.91	0.10
	Item 38	0.80	0.27
	Item 39	0.88	0.15
	Item 40*	0.84	0.18
	Item 41*	0.65	0.52
Upholding the rights of human participants	Item 42	0.89	0.12
	Item 43	0.91	0.09
	Item 44	0.94	0.07
	Item 45	0.66	0.50

*The correlation coefficient between the error variances of items 1 and 2 is 0.28, and the correlation coefficient between the error variances of items 40 and 41 is 0.40

IRB-RAT-CV = institutional review board researcher assessment tool Chinese version

**Table 3:** Standardized Factor Loadings and error variances of 45-Item IRB-RAT-CV for Actual IRB

Actual IRB			
Domain	Items	Factor-item loading	Error variances
Procedural justice	Item 1*	0.78	0.34
	Item 2*	0.86	0.21
	Item 3	0.89	0.16
	Item 4	0.91	0.14
	Item 5	0.90	0.16
	Item 6	0.87	0.19
	Item 7	0.88	0.20
Interactional justice	Item 8	0.91	0.14
	Item 9	0.91	0.14
	Item 10	0.88	0.17
	Item 11	0.93	0.12
	Item 12	0.88	0.20
Absence of bias	Item 13	0.91	0.13
	Item 14	0.91	0.15
	Item 15	0.82	0.26
	Item 16	0.91	0.14
	Item 17	0.91	0.15
Pro-science sensitivity	Item 18	0.91	0.13
	Item 19	0.92	0.13
	Item 20	0.91	0.14
	Item 21	0.90	0.16
	Item 22	0.90	0.15
IRB competence	Item 23	0.90	0.18
	Item 24	0.91	0.14
	Item 25	0.91	0.13
	Item 26	0.90	0.16
	Item 27	0.93	0.13
	Item 28	0.88	0.23
	Item 29	0.87	0.24
	Item 30	0.90	0.19
IRB outreach	Item 31	0.92	0.14
	Item 32	0.90	0.21
	Item 33	0.91	0.17
	Item 34	0.90	0.19
IRB formal functioning, structure, and composition	Item 35	0.92	0.13
	Item 36	0.89	0.17
	Item 37	0.88	0.21
	Item 38	0.85	0.27
	Item 39	0.89	0.20
	Item 40*	0.87	0.20
	Item 41*	0.76	0.44
Upholding the rights of human participants	Item 42	0.86	0.21
	Item 43	0.92	0.14
	Item 44	0.94	0.10
	Item 45	0.79	0.39

*The correlation coefficient between the error variances of items 1 and 2 is 0.35, and the correlation coefficient between the error variances of items 40 and 41 is 0.48

IRB-RAT-CV = institutional review board researcher assessment tool Chinese version

**Table 4:** Model fit indices of IRB-RAT-CV

	χ^2^	df	χ^2^/df	RMSEA	CFI	NFI	TLI
Ideal IRB	2571.825	915	2.811	0.062	0.936	0.904	0.931
Actual IRB	2714.603	915	2.967	0.065	0.941	0.914	0.936

IRB-RAT-CV = institutional review board researcher assessment tool Chinese version; χ^2^ = chi square test; df = degrees of freedom; RMSEA = root mean square error of approximation; CFI = comparative fit index; NFI = normed fit index; TLI = Tucker Lewis index

## Discussion

The main objective of this study was to validate a tool to evaluate the quality of IRBs in China. Since there is no theory-based structure or pre-validated instrument in China, we translated the IRB-RAT developed by Koocher et al. into Chinese [34]. The translation process was implemented rigorously to ensure equivalence. Because cultural and social differences between China and the West may affect IRB members’ and researchers’ understanding of IRBs, we also tested the suitability of the IRB-RAT-CV for Chinese culture. Eight experts with different roles (two IRB members, two IRB staff members, two IRB managers, and two investigators) were asked for their opinions on the framework and content of the questionnaire. At the end of this process, we retained all of the original 45 items, due to agreement among the experts consulted that the framework and content of the questionnaire was suitable. Our findings suggest that the IRB-RAT-CV is a reliable, valid tool for evaluating the quality of IRBs in the Chinese cultural context.

In terms of reliability, Cronbach's alpha coefficients exceeding 0.7 for the questionnaire as a whole and for each dimension indicated that the IRB-RAT-CV is satisfactory. All item-to-total correlation coefficients revealed high correlation with the total scale. By comparison, results from a study adapting the IRB-RAT for Peruvian culture yielded Cronbach's alpha coefficients for participants’ ideal and actual IRBs of 0.67–0.89 and 0.83–0.92, respectively [31], which are lower than the Cronbach's alpha coefficients obtained in our study. It should be noted that there may be small differences in the results obtained from these two different samples, but such differences are within the acceptable range.

Our results also suggested that the IRB-RAT-CV has good validity. Our assessment of content validity indicated that the content of the questionnaire is well-connected and well-distributed, and our assessment of structural validity indicated that the overall structure of the questionnaire is appropriate. Regarding the CFA, we adjusted the model twice. Because the CFA’s results of the original model show that items 1 and 2, and items 40 and 41 have a certain correlation, after checking the specific content of these items, we believe that there is indeed a strong correlation between these items. For example, items 1 and 2 both emphasize the IRB’s requirements for reviewing projects, and items 40 and 41 both emphasize the composition of the IRB’s membership. Therefore, we have related items 1 and 2, and items 40 and 41 on the original model.

To our knowledge, only one previous study adapting the IRB-RAT for a specific cultural context has resulted in deletion of items from the tool. The aforementioned study adapting the IRB-RAT for use in Peruvian culture used information from the internal consistency analysis to detect the most heterogeneous items of the IRB-RAT, thus generating a version that was shorter (29 items), but had better psychometric characteristics for their target population [31]. By comparison, in our study, because our Kendall’s W was greater than 0.4 (moderate), the internal consistency coefficient was greater than 0.9 (excellent), and the CFA model fitting results showed that the factor loadings of all items were greater than 0.6 (items with factor loadings greater than 0.40 are used for factor interpretation), we did not delete any items; the IRB-RAT-CV retained all of the original 45 items from the IRB-RAT. Overall, the results suggest that the IRB-RAT-CV can be used to assess the quality of IRBs in China in a consistent manner.

## Conclusions

When evaluating the quality of an IRB within a Chinese institution, as in other contexts, international evaluation standards should be combined with the local context to develop an evaluation standard suitable for the institution. This study highlights the importance of IRB evaluation as a means of promoting continuous quality improvement. The adaptation and validation of the IRB-RAT-CV tool in this study will facilitate the development of IRBs in China and further enhance the quality of IRB reviews.

In summary, the reliability and validity measures obtained by this study support the use of the IRB-RAT-CV to assess the quality of IRBs in China. Our data provides a basis for future quality evaluation studies of IRBs in China. This tool will be useful in designing additional studies to assess the relative level of development and quality of Chinese IRBs in an international context, and will aid more effective IRB quality evaluation in China.

## Study limitations

The main limitation of this study is that respondents were limited to one region of Mainland China, which may limit generalizability to other Chinese-speaking regions. This study was also limited by the use of convenience sampling, which may further limit the generalizability of findings. We chose to include researchers in our study sample rather than solely recruiting IRB staff members, in order to offset the potential bias of IRB staff members’ self-report assessment of their IRBs’ performance. However, it can be considered a further limitation of this study that the inclusion criteria were limited to IRB staff members and researchers. Research coordinators and other individuals who may have different experiences with IRBs and ethical values were not included in the present study, and may offer valuable perspectives in future studies.

## Data Availability

The datasets used and/or analysed during the current study are available from the corresponding author on reasonable request. Anyone with interest in viewing or applying the IRB-RAT-CV for research purposes, please contact corresponding author Xiaomin Wang (xiaominwang@csu.edu.cn) to request a version of the adapted tool.

## Abbreviations

IRB: Institutional Review Board
IRB-RAT-CV: Institutional Review Board-Researcher Assessment Tool-Chinese Version
ICH: The International Council for Harmonization of Technical Requirements for Pharmaceuticals for Human Use
NHC: The National Health Commission
CFDA: The China Food and Drug Administration
NMPA: National Medical Product Administration
SPSS: Statistical Product and Service Solutions
AMOS: Analysis of moment structure
CFA: Confirmatory factor analysis
ML: Maximum likelihood
RMSEA: Root mean square error of approximation
CFI: Comparative fit index
TLI: Tucker Lewis index
NFI: Normed fit index
